# Applying the Suprainguinal Approach of Fascia Iliaca Compartment Block for Surgical Anesthesia in a Patient Undergoing Emergency Femoral Thrombectomy: A Case Report

**DOI:** 10.7759/cureus.43605

**Published:** 2023-08-16

**Authors:** Eleftheria D Soulioti, Dimitrios G Antonopoulos, Dimitrios E Manikis, Ioannis D Kakisis, Paraskevi K Matsota

**Affiliations:** 1 2nd Department of Anesthesiology, Attikon University Hospital, National and Kapodistrian University of Athens, Athens, GRC; 2 2nd Department of Vascular Surgery, Attikon University Hospital, National and Kapodistrian University of Athens, Athens, GRC

**Keywords:** cardiac amyloidosis, vascular surgery anesthesia, thrombectomy anesthesia, peripheral nerve block, fascia iliaca block, suprainguinal approach, case report

## Abstract

We present the first documented case of achieving surgical anesthesia for a vascular surgery using the suprainguinal approach of the fascia iliaca compartment block (SFICB), in a patient with severe comorbidities from the cardiovascular system. More specifically, a male elderly patient with a history of cardiac amyloidosis, severe aortic stenosis, and coronary artery disease, was in need of emergent thrombectomy due to acute lower limb ischemia. During the evaluation of this patient, general and neuraxial anesthesia were both considered. However, the former would expose him to the risk of myocardial ischemia and other complications due to cardiovascular instability caused by the general anesthetic agents while the latter was absolutely contraindicated due to recent clopidogrel use and the specific pathophysiology changes induced by cardiac amyloidosis. Thus, a peripheral nerve block was deemed to be the best option in this case. SFICB, despite being challenging, could offer adequate analgesic results so it was the anesthetic technique of choice. The surgery was completed and the patient recovered appropriately. The aim of this report is to discuss the specific anesthetic considerations of this case, highlight the ability of SFICB to achieve surgical anesthesia in vascular surgeries, and increase familiarity with the procedure.

## Introduction

Acute lower limb ischemia (ALI), defined as the sudden decrease in lower limb perfusion, is a critical condition, often necessitating emergent vascular surgery [1]. The patients suffering from this condition are usually elderly with multiple comorbidities, prompting the anesthesiologist to seek the safest anesthetic technique for each case. Alternatives to general anesthesia (GA) have been utilized, including neuraxial anesthesia (NA) and peripheral nerve blocks.

The fascia iliaca is a band of connective tissue expanding from the iliac crest laterally, to the fascia overlying the psoas muscle medially. It lies anteriorly to the iliopsoas muscle, and the potential space created between these two anatomical structures is called the fascia iliaca compartment. The femoral nerve (FN), the lateral femoral cutaneous nerve of the thigh (LFCN), and the obturator nerve (ON) are all branches of the lumbar plexus, which, proximally in their anatomical course, lie within the fascia iliaca compartment [2].

The fascia iliaca compartment block (FICB), first described in 1989 [3], is a popular anesthetic technique that can offer surgical anesthesia in patients with ALI [4]. This procedure involves the injection of local anesthetic beneath the fascia iliaca in order to anesthetize the FN, LFCN, and ON. The block can even extend to the genitofemoral and ilioinguinal nerve, which are also branches of the lumbar plexus [4]. Because no nerve is directly targeted, there is a low risk of neuropraxia.

The suprainguinal approach of fascia iliaca compartment block (SFICB) was first described in 2011 [5]. Like the classic infrainguinal (IFICB) approach, it reliably blocks the FN; however, it offers higher rates of LFCN and ON blockade [6,7]. Better analgesia is provided but at the expense of a more challenging and time-consuming technique. Here, we present the first documented case of the successful application of SFICB as a primary anesthetic technique in a patient undergoing emergent femoral thrombectomy.

## Case presentation

A male patient, 85 years old, with a history of an aortobifemoral bypass surgery presented to the emergency department of our hospital with acute ischemia of his left lower limb. A computed tomography angiography was ordered, which disclosed thrombosis of the left limb of the aortobifemoral graft. His condition necessitated an urgent intervention for revascularization of the lower limb, so he was scheduled for a thrombectomy.

The pre-anesthetic evaluation of this patient revealed significant cardiovascular comorbidities. He had coronary artery disease (CAD), for which he had undergone percutaneous transluminal coronary angioplasty in the past. In addition, he was suffering from cardiac amyloidosis (CA) and severe aortic stenosis (AS), for which he had been evaluated by the heart team of the hospital and was planned to undergo transcatheter aortic valve implantation in the next months. Furthermore, the history was positive for arterial hypertension, dyslipidemia, and cigarette smoking (80 pack-years). His pharmaceutic regimen included clopidogrel, furosemide, amiloride, tafamidis, pentoxifylline, nebivolol, irbesartan, ezetimibe and atorvastatin.

No significant abnormality was diagnosed on clinical examination, including the examination of the airway. His BMI was 24.1 kg/m^2^. He was fasted for more than 24 hours. Laboratory examination was within the normal range. The coagulation profile of the patient, including the platelet count, was also normal. A recent transthoracic echocardiogram, was showing dilation of both atria, left ventricular hypertrophy, preserved ejection fraction, and severe AS (aortic valve area=0.9 cm^2^, mean gradient=29 mmHg, peak velocity=3.6 m/s, and stroke volume index=34 ml/m^2^). He was classified as American Society of Anesthesiologists' physical status grade III - emergency.

After obtaining informed consent, the patient proceeded to the operating room. Nasal oxygen was started at a flow rate of 3 l/min and two intravenous cannulas of 20G size were placed. Routine monitoring was initiated, and the left radial artery was cannulated for invasive blood pressure measurement. Cardiac resuscitation drugs were available.

**Figure 1:**
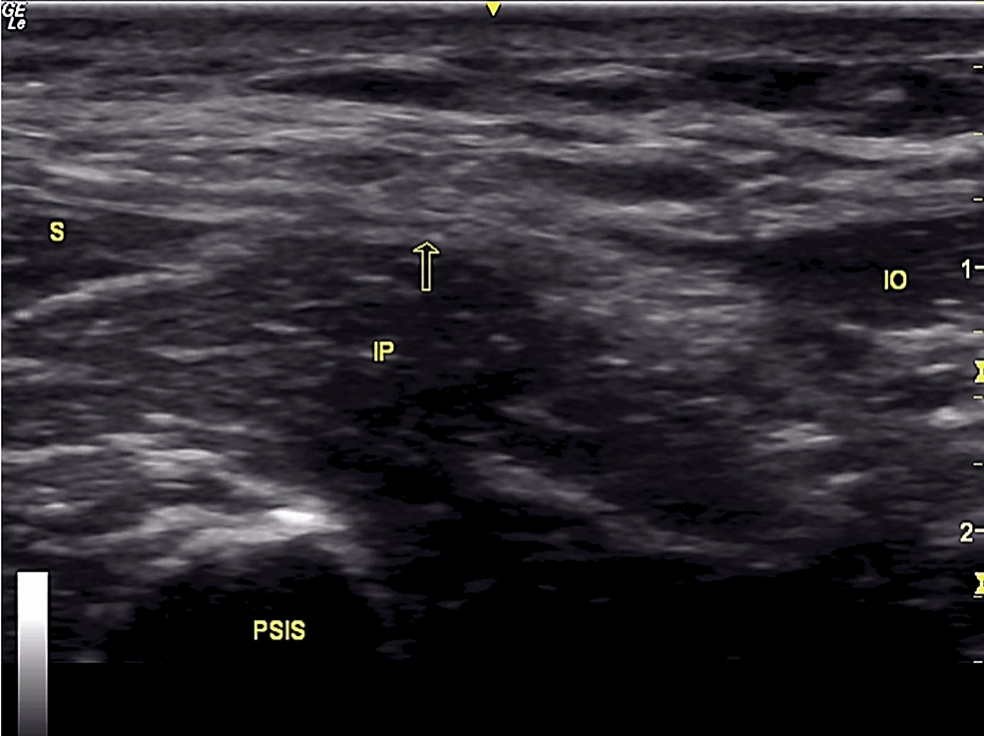
Bow tie sign S: Sartorius muscle, IO: Internal Oblique muscle, IL: Iliopsoas muscle, PSIS: Posterior Superior Iliac Spine The arrow indicates the position where ropivacaine was infused.

SFICB was performed with the patient in the supine position while the skin over the area of interest was disinfected. Using the Desmet approach, a high-frequency linear probe was placed at the level of the anterior superior iliac spine [7]. Moving the probe medially, the sartorius, internal oblique, fascia iliaca, and iliopsoas were identified. The aim was to visualize and identify the "bow tie sign" formed by muscle fasciae (Figure 1). An echogenic needle was introduced to the skin 1 cm superior to the inguinal ligament and with an "in-plane" technique, the fascia iliaca was separated from the iliacus muscle with hydro-dissection. The upward movement of the deep circumflex artery confirmed the correct needle positioning. Thirty ml of ropivacaine 0.375% was administered, and the spread was observed in the space created, superficially to the iliacus muscle. The technique was completely aseptic.

After 30 minutes, sensory levels were checked and a vertical groin incision was performed. The femoral anastomosis of the graft was exposed, along with the arteries of the left femoral bifurcation. Thrombectomy of the left limb of the graft restored appropriate inflow, and patch angioplasty of the deep femoral artery restored adequate outflow. Completion angiography confirmed the good surgical outcome.

The block was supplemented with a continuous infusion of remifentanil at a rate of 0.02 micrograms/kg/min. The patient retained a patent airway, normal respiratory rate, and hemodynamic stability throughout the surgery with a mean heart rate and systolic and diastolic pressure of 75 bpm, 120 mmHg, and 70 mmHg, respectively. No adverse events were observed and the operation was completed within two hours. The patient was transferred initially to the postanesthetic care unit and ultimately to the vascular surgery ward. No complications were observed after the surgery and tramadol at a dose of 50 mg was provided every eight hours to ease the postoperative pain until the patient was finally discharged.

## Discussion

To the best of our knowledge, this is the first report of SFICB being the primary anesthetic technique in vascular surgery. The infrainguinal approach has already been documented as capable of being the sole anesthesia technique in femoral thrombectomies [8].

There were specific considerations that prompted us to SFICB instead of other anesthetic techniques. First of all, the patient's history was positive for old age, CAD, CA, and severe AS. General anesthetic agents are known to cause severe cardiovascular instability, therefore employing GA in this case would jeopardize myocardial perfusion. Amyloid deposition in the coronary vessels, due to preexisting CA, was placing an extra burden [9]. However, one should note that these conditions are only relative contraindications of GA, thus we would be forced to proceed with it despite the high risk if no other reasonable alternatives were available.

On the other hand, NA, a well-established anesthesia technique for lower limb revascularization [10], was absolutely contraindicated due to clopidogrel use by the patient. Nonetheless, even if this was not the case, it would still be of high risk for this patient because of the specific pathophysiology changes posed by the diseases present in his medical history. More specifically, spinal and epidural infusion of local anesthetics causes inadvertent sympathetic blockade (in addition to the deliberate motor and sensory blockade), leading to peripheral vasodilation. This reduction in the circulating blood volume is detrimental in severe AS [11], in which the left ventricle hypertrophies and becomes preload dependent. Furthermore, migration of the local anesthetics in higher spinal levels blocks the sympathetic response to vasodilation-induced hypotension, causing bradycardia. This reflex is not problematic in isolated AS because the lower heart rate permits greater filling of the left ventricle, thus increasing cardiac output. However, in CA, there is amyloid deposition not only in the aortic valve (inciting the pathogenesis of AS) but also in the ventricular myocardium. This infiltration causes restrictive cardiomyopathy. The left ventricle cannot dilate appropriately and stops being preload dependent, leaving a fast heart rate as the sole mechanism for preserving cardiac output [9].

Therefore, spinal or epidural blockade in this case would cause vasodilation and bradycardia, both catastrophic for our specific patient. In general, NA is only relatively contraindicated in isolated severe AS, because it can still be performed under well-controlled conditions. However, in concomitant CA, the risk is probably increased even more. Further research is necessary to deduce if neuraxial blockades have a role in these patients.

Given all these considerations, we concluded that a peripheral nerve blockade would be the most beneficial option for this case. The choice of SFICB was totally based on the patient’s medical profile. For patients on anticoagulant or antiplatelet drugs, considering the depth of the needle’s target and the vicinity of structures at risk for hemorrhage is of major importance. Therefore, some blocks that also target the lumbar plexus nerves but carry a high risk for bleeding, such as quadratus lumborum and lumbar plexus block, should be avoided, as they could increase patient morbidity [12]. On the other hand, SFICB has a lower bleeding risk, and the use of ultrasound has effectively reduced any other serious complications like bladder perforation [5].

IFICB, which carries an even lower bleeding risk [12], had already been described as an effective technique in transfemoral thrombectomy [3]. However, there were specific reasons to presume that SFICB would achieve even better analgesia, at appropriate levels for surgical anesthesia [13]. There is extensive research regarding this approach in orthopedic surgeries. Although innervation of the femoral head from branches of the sciatic nerve prevents using SFICB as the sole anesthetic technique in total hip arthroplasty and femoral head fractures surgeries, this block is still frequently employed for pre-, peri-, or postoperative analgesia [13,14]. Data from these studies reveals that compared to the infrainguinal alternative, it causes an equally reliable blockade of the FN combined with more consistent rates of ON and LFCN blockade. This is probably explained by the direction of the needle in SFICB, which is more cephalad, allowing a better spread to the three nerves, and by the more proximal infusion of the local anesthetic targeting an area where the nerves are close to each other [6,7,10]. More specifically, both the ON and LCFN pass through the fascia iliaca and leave the fascia iliaca compartment around the level of the inguinal ligament [2]. Therefore, the cephalad instead of the transverse direction of injection and infusion above the inguinal ligament guide the local anesthetic toward not only FN but ON and LFCN as well.

Overall, the SFICB provided an effective blockade of the three nerves involved in the innervation of the area where the surgical intervention was performed while the IFICB would be less reliable. Appropriate spread to the target area was ensured by the use of an adequate volume of local anesthetic, and strong analgesia, at levels appropriate for surgical anesthesia, was achieved by the use of a relatively high concentration of ropivacaine. The block in our patient was supplemented with a continuous remifentanil infusion, so this is a potential limitation in generalizing the conclusions of this case report. However, this is a novel approach that may be of great use in patients undergoing vascular surgeries of the thigh, where other anesthetics methods are contraindicated.

## Conclusions

Expertise in regional anesthesia, along with an understanding of the pathophysiology of multiple organ system derangements, would enable the anesthesiologist to improve outcomes in managing cases with complicated comorbidities. SFICB, along with short-acting opioids like remifentanil can possibly expand the armamentarium of the techniques capable of achieving surgical anesthesia in transfemoral thrombectomy or other related operations. Further research, involving a large number of patients, is definitely necessary to determine its role and its relative advantages in vascular surgeries as compared to alternative methods.
